# The Role of Fine-Needle Aspiration Cytology in the Evaluation of Breast Lumps

**DOI:** 10.7759/cureus.70748

**Published:** 2024-10-03

**Authors:** Mohan Pradhan, Arnab Mandal, Biplab K Biswas, Anirban Hazra, Deepak Kumar

**Affiliations:** 1 General Surgery, Bankura Sammilani Medical College and Hospital, Bankura, IND; 2 Pathology and Laboratory Medicine, Bankura Sammilani Medical College and Hospital, Bankura, IND; 3 General Surgery, Calcutta National Medical College and Hospital, Kolkata, IND

**Keywords:** breast cancer, breast lump, breast swelling, ca breast, ca breast prognostic markers, core needle biopsy, fine needle aspiration cytology (fnac), fnac, histopathological diagnosis, tru-cut biopsy

## Abstract

Background

Breast cancer is the most frequently diagnosed cancer in women worldwide, accounting for more than one in ten new cancer cases each year. It ranks as the second leading cause of cancer-related mortality among women. The majority of patients present with palpable breast lumps. Effective surgical management of breast cancer largely depends on accurate preoperative pathological diagnosis. This study evaluates the diagnostic accuracy and prognostic implications of fine-needle aspiration cytology (FNAC) compared with core needle biopsy (CNB) in breast carcinoma.

Objectives

The objectives of this study are to assess the sensitivity and specificity of FNAC and CNB, to compare the diagnostic accuracy of FNAC and CNB against histopathological findings from gross specimens in the evaluation of breast lumps, and to identify and examine the limitations associated with both FNAC and CNB procedures.

Materials and methods

This study included female patients presenting with clinically suspicious palpable breast lumps at the General Surgery OPD of Bankura Sammilani Medical College and Hospital, Bankura. All patients underwent FNAC followed by CNB. The cytological and CNB diagnoses were compared with the final pathological diagnosis obtained from excisional biopsy.

Results

The study included 44 female patients aged 20 to 70 years. The most common age group for breast carcinoma was 50-59 years (36.36%). Malignancy was diagnosed in 75% of cases (33/44), with right breast involvement (65%) being more common than the left. The upper outer quadrant (59%) was the most frequently affected area. Among the 33 confirmed malignant cases, 69.70% had lesions larger than 5 cm. FNAC demonstrated a sensitivity of 93.93%, specificity of 100%, positive predictive value (PPV) of 100%, negative predictive value (NPV) of 84.61%, and diagnostic accuracy of 95.45%. CNB showed a sensitivity of 96.97%, specificity of 100%, PPV of 100%, NPV of 91.67%, and diagnostic accuracy of 97.73%. Both methods correlated significantly with the final histopathology results (p < 0.05). FNAC identified ductal carcinoma in 93.55% of cases, while CNB identified it in 96.77%.

Conclusion

CNB provides additional information on receptor status but is more resource-intensive. FNAC remains a cost-effective and time-efficient first-line diagnostic tool, especially in resource-constrained settings like rural India. FNAC should be employed for initial diagnosis, with CNB reserved for cases requiring further clarification.

## Introduction

Breast diseases encompass a broad spectrum of conditions, including inflammatory disorders, neoplastic growths, and hormonal changes. Among these, benign lesions are more frequently encountered, yet the significance of malignant breast conditions cannot be overstated. Breast cancer remains one of the most prevalent malignancies affecting women worldwide and is a leading cause of cancer-related mortality [1,2].

In India, the incidence of breast cancer is notably high, with a reported prevalence rate of 25.8 cases per 100,000 women [3]. Given the country's substantial population of approximately 1.3 billion, nearly half of whom are female, this translates into an estimated 350,000 women affected by breast cancer. This figure underscores the critical public health challenge posed by the disease, making breast cancer the most common cancer among women in India, surpassing even cervical cancer [3]. The highest rates of breast cancer are observed in major urban centers such as Thiruvananthapuram, Chennai, Delhi, and Mumbai [3]. The frequent occurrence of breast cancer in surgical practice highlights the necessity for accurate differentiation between benign and malignant lesions to ensure appropriate management [4].

Recent advancements in diagnostic techniques have led to the adoption of fine-needle aspiration cytology (FNAC) and core needle biopsy (CNB) as key methods for breast lump evaluation. These techniques offer several advantages over traditional incisional or excisional biopsies, including their suitability for outpatient settings, cost-effectiveness, avoidance of general anesthesia, and reduced patient discomfort [5].

Differentiating between benign and malignant breast lesions solely through clinical examination and ultrasonography can be challenging. Therefore, combining various diagnostic modalities is often essential. FNAC and CNB are considered complementary procedures in this diagnostic approach [5,6]. Previous studies have suggested that while there is no definitive evidence favoring one method over the other, a combination of FNAC and CNB provides a comprehensive diagnostic strategy for challenging cases [5]. FNAC is known for its rapid, cost-effective, and reliable performance, making it particularly valuable in resource-limited settings [7]. However, when FNAC results are inconclusive, CNB serves as an effective secondary diagnostic tool to reduce the risk of missed diagnoses.

This study aims to evaluate and compare the efficacy of FNAC and CNB in diagnosing breast lumps, focusing on their diagnostic accuracy and reliability. By analyzing the results from these diagnostic methods and comparing them with final postoperative histopathological findings, this study seeks to provide insights into their clinical utility and guide future diagnostic practices.

## Materials and methods

Study design

This research is a cross-sectional study conducted over 18 months, from June 2021 to November 2022. The study was carried out in the male and female surgical wards of the Department of General Surgery, in collaboration with the Department of Pathology at Bankura Sammilani Medical College and Hospital.

Period of study

The preparatory phase lasted from June 2021 to July 2021 (2 months). Data collection occurred from August 2021 to July 2022 (12 months). Data entry and analysis took place from August 2022 to October 2022 (3 months), followed by report writing in November 2022 (1 month).

Study population

The study included all female patients presenting with a clinically suspicious palpable breast lump at the General Surgery Outpatient Department (OPD) and those admitted to the surgical wards of Bankura Sammilani Medical College and Hospital. Patients were selected based on predefined inclusion and exclusion criteria.

Inclusion criteria

Female patients with a clinically palpable breast lump were included in the study.

Exclusion criteria

Patients who chose not to participate in the study, those receiving neoadjuvant therapy, patients with a breast lump not considered for surgery, ultrasonography suggestive of fibroadenosis, cystic swelling, or breast abscess, individuals with bleeding disorders, and those unwilling to undergo FNAC or CNB were excluded.

Sample size determination

The sample size was calculated using Buderer's formula [8] for diagnostic accuracy studies:

$n = \frac{z^2 \times S_N \times (1 - S_N)}{L^2 \times \text{Prevalence}}$

where n is the sample size, z = 1.96 (two-tailed at a 95% confidence interval), S_N_ is the sensitivity of FNAC as reported in a previous study (97.22%) [9], L is an acceptable error (0.1), and P is the prevalence of the target disease (25.8%) [10].

The calculated sample size was 40. Accounting for a 10% non-respondent rate, the final sample size was adjusted to 44 participants.

Procedures

Fine-Needle Aspiration Cytology

FNAC was performed as a minimally invasive procedure in which a fine needle was guided by palpation into the palpable breast lump to aspirate cells for cytological examination. The procedure was conducted under aseptic conditions using a 22-25 gauge needle attached to a 10 mL syringe. Multiple passes were made to ensure an adequate sample was obtained. The aspirated material was smeared onto glass slides, fixed with ethanol, and stained with Papanicolaou or May-Grünwald-Giemsa stain. The slides were then examined under a microscope by a pathologist to identify cytological features indicative of malignancy or benignity.

Core Needle Biopsy

CNB was performed under local anesthesia using a 14-16 gauge core biopsy needle. The procedure involved making a small incision on the skin over the lump and inserting the needle by palpating the lump to extract core tissue samples. The samples were then fixed in formalin and processed for histopathological examination. The tissue cores were examined for architectural patterns, cell morphology, and other histological features consistent with benign or malignant pathology. The average time taken to receive a CNB report from the day of sample collection ranged from 8 to 10 days, whereas the FNAC report was typically provided the following day.

Data collection and analysis

Data were collected using pre-designed Case Record Forms, capturing clinical details, FNAC and CNB results, and final histopathology findings. Statistical analysis of the collected data was performed using IBM SPSS Statistics for Windows, Version 26 (Released 2019; IBM Corp., Armonk, New York) and Microsoft Excel (Microsoft Corporation, Redmond, Washington). The evaluation of FNAC and CNB performance involved several statistical measures. Sensitivity was assessed to determine the ability of FNAC and CNB to correctly identify malignant cases, while specificity was measured to gauge their ability to correctly identify benign cases. The positive predictive value (PPV) was calculated to estimate the probability that patients with a positive test result actually had malignant lesions, and the negative predictive value (NPV) was determined to estimate the probability that patients with a negative test result did not have malignant lesions. Additionally, diagnostic accuracy was evaluated to assess the overall correctness of FNAC and CNB diagnoses in comparison to the final histopathological diagnosis. To determine the significance of correlations between the diagnostic methods and histopathological results, chi-square tests were applied, with a p-value of less than 0.05 considered statistically significant.

## Results

Patient demographics and tumor characteristics

The study included 44 female patients who presented with palpable breast lumps. The most common age groups were 40-49 and 50-59 years. Benign lesions were most frequently found in the 20-29 age group, while malignant lesions were predominantly observed in the 50-59 age group. Of the 44 cases examined, 25 patients (56.82%) had lumps larger than 5 cm. Among the 33 malignant cases, 23 patients (69.70%) had lump sizes greater than 5 cm. The TNM staging of the 33 malignant cases revealed that 16 patients (50%) were in stage IIB, followed by seven patients (21.88%) in stage IIA. Stages IIIA and IIIB each had three cases (9.38%), while stages I and IV each had two cases (6.25%). There were no cases in stage IIIC (0%).

Diagnostic accuracy of FNAC

FNAC was performed on all 44 patients. FNAC results indicated that 31 cases (70.45%) were malignant, while 13 cases (29.55%) were benign. Among the malignant cases, 29 (66%) were diagnosed as ductal carcinoma (DC), and two cases (4.5%) were diagnosed as lobular carcinoma. Among the benign cases, fibroadenoma was observed in five cases (11.36%), atypical epithelial hyperplasia in two cases (4.55%), non-specific mastitis in two cases (4.55%), fibrocystic disease in two cases (4.55%), and phyllodes tumor in two cases (4.55%).

Comparison With HPE

Table 1 shows the comparison of FNAC results with the final histopathological examination (HPE). FNAC identified all 31 malignant cases correctly (100%), but it misclassified 2 cases as benign, which were confirmed as malignant by HPE.

**Table 1:** Comparison of fine-needle aspiration cytology (FNAC) results with histopathological examination (HPE)

FNAC result	Malignant (HPE)	Benign (HPE)
Malignant (31)	31 (100%)	0 (0%)
Benign (13)	2 (15.38%)	11 (84.61%)
Total (44)	33	11

Diagnostic Performance

As illustrated in Table 2, FNAC demonstrated a sensitivity of 93.93%, specificity of 100%, positive predictive value of 100%, and negative predictive value of 84.61%. The overall diagnostic accuracy was 95.45%, with the confidence intervals as follows: 79.77% to 99.26% for sensitivity, 71.51% to 100% for specificity, and 84.53% to 99.44% for accuracy. The Fisher's exact test yielded a two-tailed P value of less than 0.0001, indicating statistical significance.

**Table 2:** Diagnostic performance of FNAC compared to HPE HPE: histopathological examination, FNAC: fine-needle aspiration cytology, CI: confidence interval.

HPE vs FNAC	95% CI
Sensitivity	93.93% (79.77% to 99.26%)
Specificity	100% (71.51% to 100%)
Positive predictive value	100%
Negative predictive value	84.61% (58.94% to 95.47%)
Accuracy	95.45% (84.53% to 99.44%)

Diagnostic accuracy of CNB

CNB was also performed on all 44 patients. The CNB results indicated malignancy in 32 cases (72.73%) and benign conditions in 12 cases (27.27%). CNB diagnosed 30 cases (68%) as ductal carcinoma and 2 cases (4.5%) as lobular carcinoma.

Comparison With HPE

Table 3 presents a comparison of CNB results with the final HPE diagnosis. CNB correctly identified 32 malignant cases (100%); however, one case that was diagnosed as benign by CNB was confirmed as malignant by HPE.

**Table 3:** Comparison of CNB results with HPE CNB: core needle biopsy, HPE: histopathological examination.

CNB Result	Malignant (HPE)	Benign (HPE)
Malignant (32)	32 (100%)	0 (0%)
Benign (12)	1 (8.33%)	11 (91.66%)
Total (44)	33	11

Diagnostic Performance

As detailed in Table 4, CNB demonstrated a sensitivity of 96.97%, specificity of 100%, positive predictive value of 100%, and negative predictive value of 91.67%. The overall diagnostic accuracy was 97.73%, with confidence intervals of 84.24% to 99.92% for sensitivity and 87.98% to 99.94% for accuracy. The Fisher's exact test provided a two-tailed P value of less than 0.0001, confirming the statistical significance of the results.

**Table 4:** Diagnostic performance of CNB compared to HPE CNB: core needle biopsy, HPE: histopathological examination.

HPE vs CNB	95% CI
Sensitivity	96.97% (84.24% to 99.92%)
Specificity	100% (71.51% to 100%)
Positive predictive value	100%
Negative predictive value	91.67% (61.49% to 98.70%)
Accuracy	97.73% (87.98% to 99.94%)

Comparison between FNAC and CNB

When comparing FNAC and CNB, both diagnostic methods demonstrated high accuracy in identifying malignant lumps, but CNB had a slight edge in sensitivity and negative predictive value. Table 5 presents the direct comparison between FNAC and CNB.

**Table 5:** Comparison between FNAC and CNB results CNB: core needle biopsy, HPE: histopathological examination.

FNAC result	CNB result	Total
Malignant (31)	31 (100%)	0 (0%)
Benign (13)	1 (7.6%)	12 (92.3%)
Total (44)	32	12

Diagnostic Performance Comparison

As shown in Table 6, FNAC had a sensitivity of 96.88%, while CNB had a sensitivity of 96.97%. Both tests exhibited a specificity of 100%, with FNAC having a positive predictive value of 100% and a negative predictive value of 92.31%. The overall accuracy of both tests was 97.73%, and Fisher's exact test yielded a two-tailed P-value of less than 0.0001.

**Table 6:** Diagnostic performance comparison between FNAC and CNB CNB: core needle biopsy, HPE: histopathological examination.

CNB vs FNAC	95% CI
Sensitivity	96.88% (83.78% to 99.92%)
Specificity	100% (73.54% to 100%)
Positive predictive value	100%
Negative predictive value	92.31% (63.55% to 98.80%)
Accuracy	97.73% (87.98% to 99.94%)

Tumor type identification

According to the FNAC report, ductal carcinoma (DC) was the most common diagnosis, observed in 29 cases (65.91%). Fibroadenoma followed with five cases (11.36%), while lobular carcinoma (LC), atypical epithelial hyperplasia, non-specific mastitis, fibrocystic disease, and phyllodes tumor were each found in two cases (4.55%). In contrast, the CNB report showed that DC was the most frequent diagnosis, with 30 cases (68.18%). Fibroadenoma was noted in five cases (11.36%), while LC, fibrocystic disease, and phyllodes tumor each appeared in two cases (4.55%). Atypical epithelial hyperplasia, granulomatous mastitis, and non-specific mastitis were each observed in one case (2.27%). Lastly, the final histopathology (HPE) report revealed that invasive ductal carcinoma (IDC) was predominant, with 31 cases (70.45%). Fibroadenoma was seen in five cases (11.36%), and LC, fibrocystic disease, and phyllodes tumor each occurred in two cases (4.55%). Granulomatous mastitis and non-specific mastitis were each identified in one case (2.27%). Notably, FNAC misdiagnosed granulomatous mastitis as non-specific mastitis, whereas CNB accurately identified it as granulomatous mastitis. This finding underscores CNB's superiority in distinguishing specific types of breast malignancies.

Surgical management

Regarding surgical management, the majority of patients underwent modified radical mastectomy (MRM), with 31 out of 44 patients (70.45%) undergoing this procedure. Additionally, toilet mastectomy was performed in two cases (4.55%). Lumpectomy, excision, and enucleation were performed in 11 cases (25%), grouped under the category of “lumpectomy” for ease of comparison.

## Discussion

Breast cancer remains the most prevalent malignancy among women globally [2]. Early detection and appropriate management are vital for achieving optimal outcomes. In developing countries, where awareness and breast screening practices are limited, patients often present with more advanced stages of breast cancer compared to developed countries, where screening programs facilitate earlier detection [7]. The triple assessment protocol, comprising clinical breast examination, imaging (mammogram/sonogram), and pathological examination (FNAC/CNB), is a standard approach. However, FNAC alone is not always sufficient for comprehensive treatment decision-making due to its limitations in providing critical information such as tumor histopathological type, grade, and receptor status, which are crucial for preoperative evaluation and surgical planning [7]. 

Study population and tumor characteristics

Our study included 44 female patients with clinically suspicious breast lumps, who underwent both FNAC and CNB, with the final pathological diagnosis from excision specimens serving as the gold standard. The results indicated that breast cancer incidence increased with age, notably in the 40-49 and 50-59 age groups. Benign lesions were most common in the 20-29 age group (45.45%), while malignant lesions were prevalent in the 50-59 age group (36.36%). These findings are consistent with Khemka et al. [11], who reported a peak incidence between 40-44 years.

Tumor size and presentation

Our study observed that 25 cases (56.82%) had lumps larger than 5 cm, with 23 (69.70%) being malignant. This is in line with Saha et al. [12], who found 64.3% of malignant lesions in lumps ranging from 5 to 10 cm. The larger size of tumors in our study likely results from delayed presentation due to a lack of awareness.

Diagnostic accuracy of FNAC

Our study results were compared with those of other studies, as demonstrated in Table 7. FNAC in our study demonstrated a sensitivity of 93.93%, which is higher than the 69% reported by Saha et al. [12] and slightly lower than the 97.22% reported by Rahman et al. [7], indicating a strong performance in detecting malignancy within our patient cohort. The specificity was perfect at 100%, consistent with other studies, underscoring FNAC's reliability in accurately identifying benign cases. The positive predictive value (PPV) of FNAC was also 100%, reaffirming its robustness in confirming malignant diagnoses. However, the negative predictive value (NPV) of 84.61% suggests a relatively lower rate of correctly identifying non-malignant cases compared to the higher NPV seen in some studies. This variation could stem from differences in study populations, the prevalence of malignancy, or slight methodological differences, such as the skill level of the cytopathologist or the quality of the cytological samples. Additionally, the slightly lower NPV highlights the potential for false negatives, emphasizing the need for cautious interpretation of negative FNAC results, particularly in clinically suspicious cases.

**Table 7:** Results of FNAC compared to final histopathology report in other study series FNAC: fine-needle aspiration cytology, HPE: histopathological examination, NA: not available.

Results of FNAC compared to final HPE	This study	Saha et al. [12]	Yusuf et al. [13]	Rahman et al. [7]
Sensitivity	93.93%	69%	81%	97.22%
Specificity	100%	100%	99%	99.46%
Positive predictive value	100%	100%	97.7%	97.22%
Negative predictive value	84.61%	38.1%	90.8%	99.46%
Accuracy	95.45%	74%	NA	99.095%

Diagnostic accuracy of CNB

Our study results were compared with those of other studies, as demonstrated in Table 8. CNB in our study exhibited a sensitivity of 96.97%, which is higher than the 88.3% reported by Saha et al. [12] and similar to that reported by Bdour et al. [13]. The specificity was perfect at 100%, consistent with other studies. The positive predictive value of 100% and a negative predictive value of 91.67% indicate that CNB is highly reliable in confirming malignancy and ruling out non-malignancy. The high sensitivity and accuracy of CNB highlight its effectiveness in diagnosing breast carcinoma.

**Table 8:** Results of CNB compared to final histopathology report in other study series CNB: core needle biopsy, HPE: histopathological examination, NA: not available.

Results of CNB compared to final HPE	This study	Saha et al. [12]	Bdour et al. [14]	Tikku et al. [15]
Sensitivity	96.97%	88.3%	97%	95.8%
Specificity	100%	100%	100%	100%
Positive predictive value	100%	100%	NA	100%
Negative predictive value	91.67%	53.8%	NA	94.9%
Accuracy	97.73%	86%	97%	NA

Comparative analysis and clinical implications

Both FNAC and CNB showed statistically significant correlations with confirmatory HPE of excision specimens (p-value <0.05) in diagnosing breast carcinoma. Statistical analysis using Fisher’s exact test between FNAC and CNB results revealed a significant correlation (p-value <0.05), confirming their diagnostic relevance.

When comparing FNAC and CNB, CNB demonstrated higher sensitivity, positive predictive value, and diagnostic accuracy compared to FNAC. The lower false-negative rate of CNB suggests it is more reliable for detecting malignancy. Both procedures showed similar specificity, indicating comparable performance in identifying benign cases. The statistically significant correlation with confirmatory HPE highlights the importance of both diagnostic methods in breast cancer diagnosis, with CNB offering a slight advantage over FNAC.

Role of FNAC in resource-limited settings

FNAC remains a valuable diagnostic tool, especially in resource-constrained settings. It is cost-effective, easily performed, and provides quick results, which is crucial in areas where CNB may not be readily available [7]. In such contexts, FNAC's ability to quickly and accurately identify benign lesions and provide a preliminary diagnosis of malignancy can guide treatment decisions effectively. While FNAC may have limitations, such as a higher false-negative rate, its role in diagnosing breast lumps and facilitating early intervention cannot be overlooked. In resource-poor countries, where CNB and advanced hormonal studies may not be accessible, FNAC remains a practical and essential option for initial cancer detection and management.

Limitations of the study

Several limitations of our study should be acknowledged. First, the small sample size of 44 participants limits the generalizability of the findings and may not fully represent the broader population. Secondly, being a single-center study, the results may not be applicable to other regions with different demographic characteristics or healthcare infrastructures. Additionally, the study did not incorporate a detailed comparison with radiological findings, which could have provided further insights into the diagnostic accuracy of FNAC and CNB. Furthermore, the lack of long-term follow-up data means that the impact of these diagnostic methods on treatment outcomes and survival rates was not assessed.

## Conclusions

In developing countries like India, where healthcare resources are limited, FNAC remains a vital tool for the early detection of breast cancer. Its simplicity, cost-effectiveness, and ease of use make it especially valuable in rural and underserved areas where advanced diagnostic tools like CNB and hormonal studies may not be available. FNAC offers a rapid and minimally invasive method for diagnosing breast lesions, providing timely and reliable results without requiring complex infrastructure.

While CNB provides detailed histopathological and receptor status information, FNAC’s practicality and lower cost make it a preferable choice in resource-constrained settings. Although CNB has slightly higher sensitivity, FNAC effectively identifies both benign and malignant lesions, reducing the need for more invasive procedures. In resource-poor countries, FNAC provides an essential option for initial cancer detection and management, allowing for prompt and efficient treatment in the absence of advanced diagnostic facilities. As healthcare systems develop, FNAC will continue to play a crucial role in enhancing breast cancer care in low-resource environments.
